# Supplementary material for: Structural insights into ubiquitin chain cleavage by Legionella ovarian tumor deubiquitinases
Source: Life Sci Alliance. 2023 Apr 26;6(7):e202201876. doi: 10.26508/lsa.202201876 (PMC10133868; doi:10.26508/lsa.202201876)
Supplement: Supplementary file 2 [file LSA-2022-01876_TableS1.docx]

**Supplementary information**

**Supplementary tables**

**Supplementary Table 1. Cryo-EM data collection and processing statistics**

|  | LotA_7-544_ |
| --- | --- |
|  | (EMD-34350) |
| *Sample Preparation* |  |
| Grid type | Quantifoil Au 1.2/1.3 200 mesh |
| Vitrification instrument | Vitrobot Mark IV |
|  |  |
| *Data collection* |  |
| Microscope | Glacios |
| Detector | Falcon 4 |
| Collection mode | Electron counting mode |
| Voltage (kV) | 200 |
| Magnification | 150 kx |
| Pixel size (Å) | 0.68 Å |
| Total electron dose (e^-^/Å^2^) | 60.14 |
| Dose rate (e^-^/Å^2^/s) | 10.59 |
| Defocus range (μm) | - 1.0 – - 1.8 |
| Exposure time (s) | 5.68 |
|  |  |
| *Initial data processing* |  |
| Processing program | *CryoSPARC* |
| Number of micrographs | 2,538 |
| Number of micrographs for processing | 1,796 |
| Total frame per each micrograph | 60 |
| Initial particle number | 498,860 |
| Selected particle number | 176,036 |
|  |  |
| *Final data processing* |  |
| Processing program | *RELION* |
| Selected particle number | 113,381 |
| Resolution (Å) | 11 |
